# Unrecognised Unilateral Blindness

**Published:** 1918-02-09

**Authors:** 


### SOME INTERESTING EYE CONDITIONS.

### Unrecognised Unilateral Blindness.

To those who have experience in the treatment of diseases of the eye it is an established conclusion that a patient's judgment on the efficiency of his eyesight is often a judgment of comparatively little value. The reason for this is obvious. He has never had but one pair of eyes, and hence has enjoyed no opportunity of making comparisons. Assuming, therefore, that his eyesight has always been defective, he has no standard by which he can recognise this defect. His only standard is his own vision, and as this may well satisfy practical purposes he accepts it as the normal level. It is indeed astonishing to find how frequently a person may during many years be the victim of very defective vision and yet be quite unaware of this defect. Sometimes the discovery is made in the course of a medical examination. At other times it results from circumstances which bring the individual's vision into competition or comparison with the vision of some of his fellows, as, for example, in shooting at a target or at game, or in the identification of some distant object. But in the absence of such experiences a person may for years, and probably indeed throughout life, remain quite unaware of the fact that his capacity for sight is far below the average level. This truth might be illustrated by many examples, but perhaps the most impressive instances are those in which a person is blind in one eye from birth, and yet only makes the discovery comparatively late in life. Were the facts not quite beyond challenge it would seem incredible that over many years such a person can have failed to close or to cover up his good eye in a fashion which would have revealed the defect of the opposite one. Yet so it is as a matter of fact, and it is therefore not surprising that in such circumstances the patient should believe with sincere conviction that his blindness is, when at last discovered, some new development. The experience may sometimes have serious consequences. Here is an instance. A man of forty-five years was rather seriously shaken in a railway accident. During convalescence he found more difficulty in reading small print than he had previously experienced, and this led him in an amateur fashion to test his eyesight, when he found to his surprise and alarm that his left eye was quite blind. Naturally he came to the conclusion that the accident had produced some serious damage to his eyesight, and on this conclusion he framed his claim for damages. Yet examination showed that while his right eye was perfectly normal there was old-standing and extensive disease in the deeper parts of the left eye, and that this eye had been blind for years, and in all probability from birth. What had happened was that the man had arrived at the age when the normal eye begins to need the help of a lens for near work. The shock of the accident and the strain of the reading which he naturally pursued while confined to bed made him conscious, for the first time, of some little difficulty in the use of his eyes. Thus his attention was directed to his eyesight and led to the discovery of his unilateral blindness. That he should hold strong views on the responsibility of the accident for the defect is not surprising. Yet it is certain that the real explanation is the one here given, and thus the case shows the possibility of an intelligent and fairly observant man having one eye completely blind and yet failing to discover the defect until a railway accident at forty-five years of age forced upon him an attitude of criticism relative to his own powers and defects. Similar experiences are not so very uncommon.

Another working rule for those engaged in ophthalmic practice is that an inflammation limited to one eye should always lead to a suspicion of the presence of a foreign body. Only when by a careful search has this possibility been excluded is it safe to fall back upon some alternative explanation. In no circumstances can this rule be omitted without risk. Here is an example in which the conditions seemed to make a relaxation of such a demand justifiable. But the event showed the rule to be essential. A patient suffered for some days from a fairly severe attack of "muscular rheumatism." In its course he developed pain and inflammation in his left eye. Not very unnaturally the practitioner concluded that these manifestations were due to "rheumatic iritis," and accordingly prescribed some atropine drops. Though these gave some relief the eye did not sensibly improve. Then when an inspection was made with a powerful lens under oblique illumination there was found imbedded in the cornea a minute fragment of dust, and the removal of this with the help of a little cocaine and a fine spud, followed by the atropine drops and a pad and bandage, promptly got rid of the "rheumatic iritis" and reduced the diagnosis to the homely proportions of a foreign body.

Two very extraordinary eye conditions have recently been reported in the journals. One, recorded in the *Lancet* of January 19, is of the presence of eyelashes in the anterior chamber without any evidence of penetration of the eyeball by a foreign body. The patients have been soldiers who complained of pain and photophobia after the explosion of a projectile, but without evidence of physical injury. There is only one possible treatment—namely, to open the anterior chamber and to remove the eyelashes. But how these latter could get through the cornea into the anterior chamber remains a mystery. Another odd experience is related by Dr. Lewis McMillan in the January issue of the *Journal of Ophthalmology*. A man attending the hospital as an out-patient exhibited his capacity to evert voluntarily one or both upper eyelids. Closing his mouth and eyes, and fixing, as it were, the lower facial muscles, "he seemed to force the lower lid under the upper lid and so levered the latter upwards into a position of eversion." He had learned the trick in childhood and had practised it since. The case is a curious illustration of the muscular dexterities which may be developed by resolution and practice.

